# Combining machine learning and simulations of a morphologically realistic model to study modulation of neuronal activity in cerebellar nuclei during absence epilepsy

**DOI:** 10.1186/1471-2202-15-S1-P39

**Published:** 2014-07-21

**Authors:** Parimala Alva, Lieke Kros, Oscar H J Eelkman Rooda, Chris I De Zeeuw, Rod Adams, Neil Davey, Freek E Hoebeek, Volker Steuber

**Affiliations:** 1 Science and Technology Research Institute, University of Hertfordshire, Hatfield AL10 9AB, UK; 2 Department of Neuroscience, Erasmus Medical Center, Rotterdam, The Netherlands; 3 Netherlands Institute for Neuroscience, Royal Dutch Academy for Arts and Sciences, Amsterdam, Netherlands

## 

Epileptic absence seizures are characterized by synchronized oscillatory activity in the cerebral cortex that can be recorded as so-called spike-and-wave discharges (SWDs) by electroencephalogram. Although the cerebral cortex and the directly connected thalamus are paramount to this particular form of epilepsy, several other parts of the mammalian brain are likely to influence this oscillatory activity. We have recently shown that some of the cerebellar nuclei (CN) neurons, which form the main output of the cerebellum, show synchronized oscillatory activity during episodes of cortical SWDs in two independent mouse models of absence epilepsy [1]. The CN neurons that show this significant correlation with the SWDs are deemed to “participate” in the seizure activity and are therefore used in our current study designed to unravel the potential causes of such oscillatory firing patterns.

**Figure 1:**
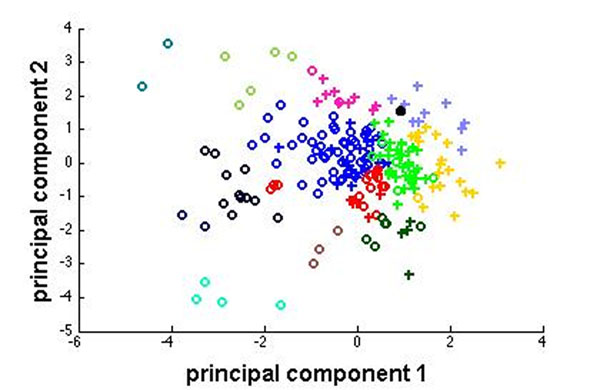
shows 2D projection, using principal component analysis, of the clusters formed as a result of GNG clustering of the CN neuron interictal activity using CV, Log-Interval Entropy, Firing rate and Permutation Entropy. The crosses (+) indicate cells that participate in the seizure and (o) indicate the cells that do not participate based on the measures, FFT based Z-score and modulation frequency. The black dot indicates the output from the computer simulations

Initially, we set out to study if different types of CN neurons are more prone to show modulated firing patterns during seizure activity. We applied Growing Neural Gas (GNG) [2], an unsupervised clustering algorithm, on the interictal activity, i.e., firing patterns recorded in between seizures, using the measures CV, log-interval entropy, permutation entropy and firing rate. Three main groups of neurons were found by the clustering algorithm, in which the neurons were predominantly participating in the seizures. These can be seen on Figure 1 as the green, yellow and pale blue crosses (+). Moreover, these three clusters have the highest CV (and therefore more irregular) and higher log-interval entropy (more unpredictable). Further, we used a Gaussian Process Regression model [3] to predict the extent of participation of the neurons in the seizure activity, based on two measures: Z-score of mean power at seizure frequency (6-9Hz) (FFT based Z-score) and modulation frequency. These characterize the extent to which CN neurons phase-lock their spiking activity to the spikes in the EEG during seizures. We achieved a good prediction rate (*r* = 0.56, *p* < 0.05 for FFT based Z-score; *r* = 0.45, *p* < 0.05 for modulation frequency) using this method. Also, we are using a compartmental model of a CN neuron with realistic morphology [4] to investigate the input conditions that can generate interictal activity found in the participating neurons. Our results indicate that bursting in the Purkinje cell or mossy fiber input can cause behavior that is similar to the interictal activity found in participating neurons. The black dot in Figure 1 shows the output from the CN neuron model, provided with a bursting Purkinje cell input, when it is subjected to clustering with the experimental data. Desynchronization of the burst occurrence in the input did not alter the position of the data point drastically[0]. Currently, we are in the process of applying an evolutionary algorithm to explore in detail the input conditions that can that lead to the spiking behavior that is associated with seizures.
